# Great Toe-to-Thumb Hemi-Pulp Transfer

**Published:** 2016-08-05

**Authors:** Haripriya S. Ayyala, Alexandra Condé-Green, Ramazi Datiashvili

**Affiliations:** Division of Plastic Surgery, Department of Surgery, Rutgers–New Jersey Medical School

**Keywords:** free tissue transfer, hand surgery, microsurgery, reconstruction, thumb

## DESCRIPTION

**Figure 1:**
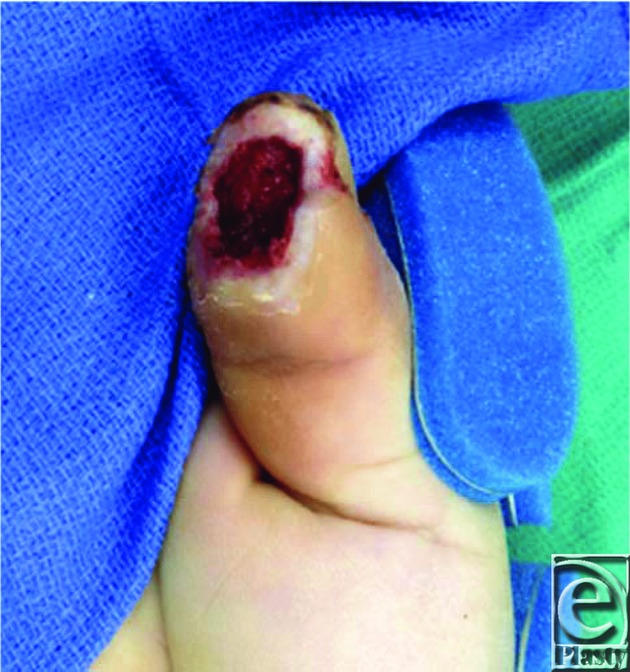
Full-thickness defect of the volar aspect of the right distal thumb.

A 56-year-old right-hand-dominant man sustained an avulsion injury to his right thumb while operating a rotating chain machine, resulting in a full-thickness traumatic defect of the volar aspect of the distal thumb measuring 7 cm^2^ (Fig 1).

## QUESTIONS

1. **What is the anatomy and function of thumb pulp tissue?**
2. **What are the goals of reconstruction?**
3. **What are the available reconstructive options?**
4. **What is the toe-to-thumb pulp transfer?**

## DISCUSSION

The thumb provides up to 50% of hand function and is vital to pinch, grasp, and grip strength. The distal volar thumb soft tissue is composed of a thick epidermis with deep papillary ridges, below which lies fibrofatty tissue stabilized by fibrous septa that extend from the periosteum of the distal phalanx to the dermis. This thumb pulp contributes more than 50% of the fingertip volume and is key to precision grip and 3-point pinch technique, modalities that are required for the completion of many activities of daily living.^1^

Defects of the volar thumb pulp are functionally limiting and should be ideally reconstructed according to the plastic surgery adage, replacing “like with like” tissue. The goals of reconstruction are to restore functional length of the thumb, provide robust coverage and sensibility, prevent contracture, and strive for the best cosmetic outcome. Ideally, the substituted tissue must also provide a match in terms of thin subcutaneous fat, texture, and strength.

There are many reconstructive options available, classified by the size of the defect. Small defects of the thumb tip are tolerated in most patients, and sensate soft-tissue coverage is the most important goal. For injuries less than 1 cm^2^, healing by secondary intention is a viable option.^2^ For defects without exposed bone or tendon, a skin graft is another option, albeit one that sacrifices prior sensation and bulk. For a volar defect of up to 1.5 cm^2^, a neurovascular advancement flap, originally described by Moberg, provides sensibility and coverage. This flap is advanced while the thumb interphalangeal (IP) joint is flexed; however, it may result in flexion contracture at the IP joint without adequate therapy.^3^^,^^4^ An alternative is the neurovascular island flap. While it transfers innervated tissue from the glabrous skin of the long finger, there is significant donor site morbidity, there are reports of venous congestion and cold intolerance, and it provides less grip and pinch sensitivity than pulp skin.^5^ For a volar defect of more than 3 cm^2^, the first dorsal metacarpal artery (FDMA) flap is a versatile flap based on a branch of the radial artery that can be transferred with cutaneous sensory branches of the radial nerve and is usually designed on the dorsum of the proximal phalanx of the index finger.^6^^,^^7^ However, the pedicle length is sometimes an issue, and the dorsal skin does not offer the same sensibility as volar glabrous skin.^5^ When these local and regional flaps are inadequate or unavailable, free tissue transfer, such as the great toe hemi-pulp transfer, should be considered.^8^

**Figure 2:**
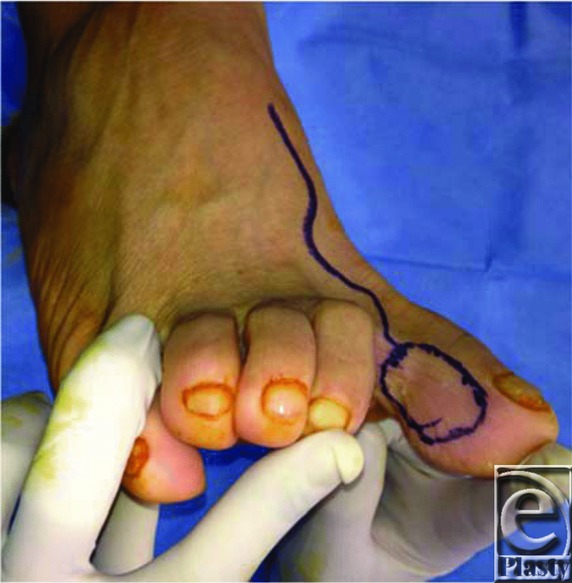
Great toe hemi-pulp flap and pedicle marking.

**Figure 3:**
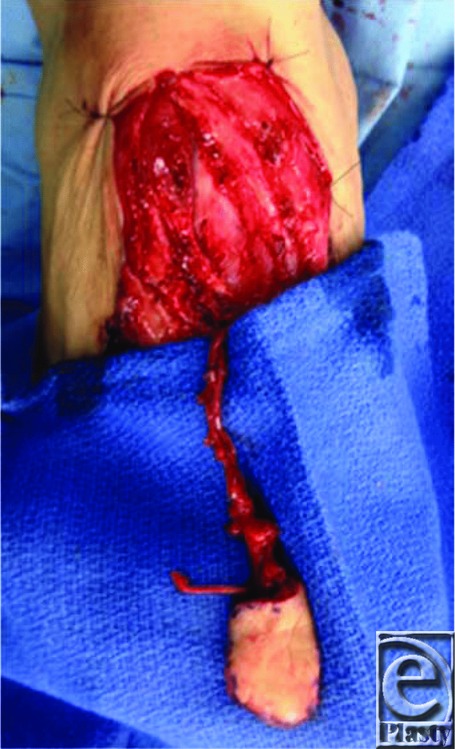
Great toe hemi-pulp flap skin paddle and pedicle.

**Figure 4:**
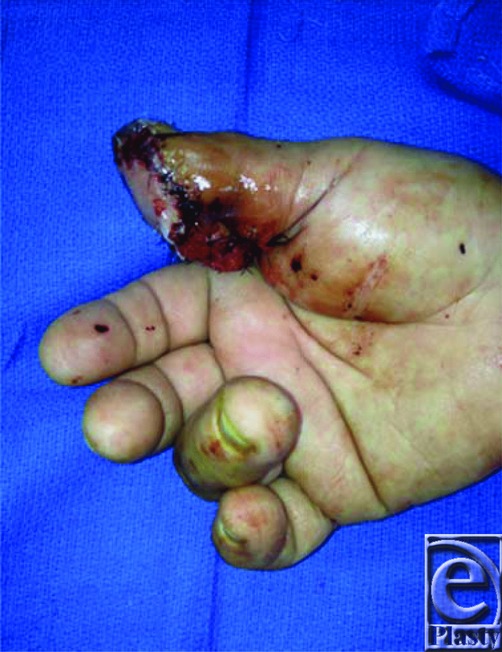
Immediate postoperative view of the great toe hemi-pulp flap.

**Figure 5:**
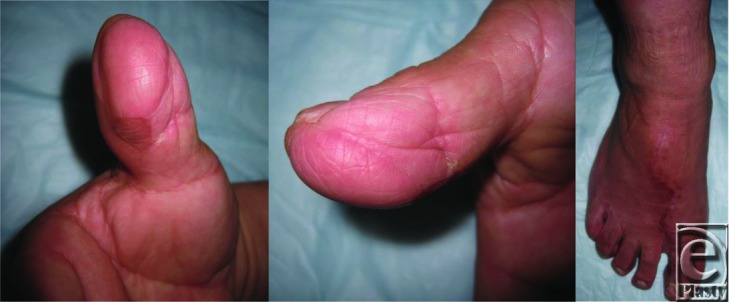
Postoperative week 10 of the great toe hemi-pulp flap and donor site.

Our patient presented with a 3.5 x 2.5-cm defect of his right thumb pulp. We considered the options discussed earlier and decided that a free neurotized flap from the right lateral great toe would be the appropriate option. It would match the texture and skin type of the defect and offer this patient the best functional outcome. We had also considered using the FDMA flap; however, the pedicle would not have been of sufficient length. The free flap pedicle was marked utilizing a Doppler scan (Fig 2); it consisted of the first dorsal metatarsal artery, an accompanying branch of the great saphenous vein, and a branch of the plantar digital nerve (Fig 3). The recipient vessels used were the radial artery in the anatomic snuffbox (end-to-side anastomosis) and the cephalic vein. The donor digital nerve was anastomosed to the ulnar digital nerve of the thumb. The recipient site was closed primarily excluding the distal portion of the vascular pedicle, which was covered with Biobrane to prevent compression of the vascular pedicle (Fig 4) and later replaced with a full-thickness skin graft from the right medial forearm. The donor site on the foot was closed primarily. Both sites healed appropriately with minimal donor site morbidity (Fig 5). Restoration of tactile sensation over the flap was observed with 2-point discrimination of 10 mm at 4 months postoperatively. Although technically demanding, there are several key advantages to the use of the toe-to-thumb pulp transfer, also known as the first web space flap. This option provides sensate, glabrous skin with the potential for restoration of sensation. This flap has a consistent pedicle, and the donor site can be closed primarily or with a split-skin autograft with minimal donor site morbidity.

The thumb is the most vital digit and contributes a great deal to hand function. There are many reconstructive options for distal volar thumb defects. While local and regional flaps are acceptable options, free great toe hemi-pulp transfer should be considered as the treatment of choice as it offers good vascularity, sensibility, and the best functional and aesthetic outcomes.
